# Clopidogrel Influences Fracture Healing Under Ischemic Conditions

**DOI:** 10.3390/biomedicines13092286

**Published:** 2025-09-17

**Authors:** Sebastian Schreiber, Janine Stutz, Lukas Keller, Wolfgang Metzger, Tobias Fritz, Christian Schönbeck, David Osche, Marcus Örgel, Michael D. Menger, Tim Pohlemann, Emmanouil Liodakis, Matthias W. Laschke, Marcel Orth

**Affiliations:** 1 Department of Trauma, Hand and Reconstructive Surgery, Saarland University, D-66421 Homburg, Germany; stutz.janine@t-online.de (J.S.); l.keller1998@t-online.de (L.K.); johann-wolfgang.metzger@uks.eu (W.M.); tobias.fritz@uks.eu (T.F.); christian_schoenbeck@icloud.com (C.S.); david.osche@uks.eu (D.O.); marcus.oergel@uks.eu (M.Ö.); tim.pohlemann@uks.eu (T.P.); emmanouil.liodakis@uks.eu (E.L.); marcel.orth@uks.eu (M.O.); 2 Institute for Clinical and Experimental Surgery, Saarland University, PharmaScienceHub (PSH), D-66421 Homburg, Germany; michael.menger@uks.eu (M.D.M.); matthias.laschke@uks.eu (M.W.L.)

**Keywords:** clopidogrel, ischemia, fracture healing, mouse, BMP-4, CD31

## Abstract

**Background/Objectives**: Patients suffering from fractures are often treated with clopidogrel during the phase of bone healing due to multiple comorbidities. Studies indicate that clopidogrel suppresses osteoblast proliferation and the formation of trabecular bone. However, it is unknown whether clopidogrel also affects fracture healing under ischemic conditions, as they may occur in multimorbid patients. **Methods**: To test this in the present study, a murine ischemia model was performed in CD-1 mice by ligating the right deep femoral artery to induce mild ischemia of the right lower limb. A closed fracture of the femur was then stabilized by inserting an intramedullary lag screw. The animals received either 3 mg/kg body weight clopidogrel daily per os or vehicle (control). Bone healing was assessed by biomechanical, radiological, histomorphometrical and Western blot analyses 2 and 5 weeks postoperatively. **Results**: The fractured femurs in the clopidogrel group exhibited no increase in biomechanical stiffness throughout the observation period in contrast to controls. While the radiological analysis showed no differences between both groups, histomorphometric analyses demonstrated a significantly reduced bridging score, less bone and more connective tissue within the callus of clopidogrel-treated animals. Western blot analyses revealed a significantly reduced expression of the osteogenic marker bone morphogenetic protein (BMP)-4 and an increased expression of the blood vessel marker CD31. **Conclusions**: These results show that clopidogrel may impair fracture healing under challenging ischemic conditions, which is associated with a shift in angiogenic and osteogenic expression markers in the callus tissue. Therefore, clopidogrel treatment may not be recommended in fracture patients with tissue ischemia.

## 1. Introduction

Clopidogrel is used in clinical practice to prevent atherothrombotic events [1,2,3]. It is applied as monotherapy for the prophylaxis of apoplexy recurrence, following a recent myocardial infarction, or in cases of existing peripheral arterial occlusive disease (PAD). In patients with critical limb ischemia (CLI), clopidogrel is recommended to reduce the risk of arterial occlusion [4,5]. Clopidogrel inhibits platelet aggregation by blocking the P_2_Y_12_ purinergic receptor on platelets, which is also expressed on osteoblasts and osteoclasts [6]. Moreover, platelets are involved in the initial inflammatory reaction to bone injury, which is crucial for the long-term outcome of bone repair [7].

Of interest, preclinical studies indicate that clopidogrel can alter the platelet function and, thereby, potentially affects bone tissue architecture and postoperative healing [6,8]. Clopidogrel has been shown to suppress osteoblast proliferation in vitro and to significantly reduce trabecular bone formation in vivo [8]. In contrast, a previous study showed in a rabbit calvarial defect model that perioperative treatment with clopidogrel does not impair bone healing, but promotes bone regeneration and defect bridging [6].

Patients with ischemia, as in PAD, tend to have lower bone mineral density [9]. Moreover, ischemia is a common co-morbidity in individuals suffering from fractures [10]. In fact, the use of clopidogrel increased by 5.8% between 2005 and 2018 [11]. Such conditions may negatively influence the highly orchestrated process of bone healing [12]. To the best of our knowledge, the effect of clopidogrel on bone healing under ischemic conditions remains to be elucidated. Therefore, the aim of the present study was to investigate the effect of clopidogrel on the process of bone healing under ischemic conditions in a well-established murine fracture model [13] by means of biomechanical, radiological, histomorphometric and Western blot analyses.

## 2. Materials and Methods

### 2.1. Animals

In the present study, a total of 46 CD-1 mice (21 males and 25 females) were used. The animals exhibited an average body weight of 39.0 ± 0.9 g. The breeding was conducted at the Institute for Clinical and Experimental Surgery, Saarland University, Germany. They were kept under a regular light and dark cycle (12-h day-night rhythm) with free access to tap water and standard pellet food (Altromin, Lage, Germany). The study was carried out in compliance with the German legislation on the protection of animals and the National Institutes of Health (NIH) Guidelines for the Care and Use of Laboratory Animals. It was approved by the local authorities (permission number: 35/2020; State Office for Consumer Protection, Saarbrücken, Germany).

### 2.2. Surgical Procedure

**Figure 1:**
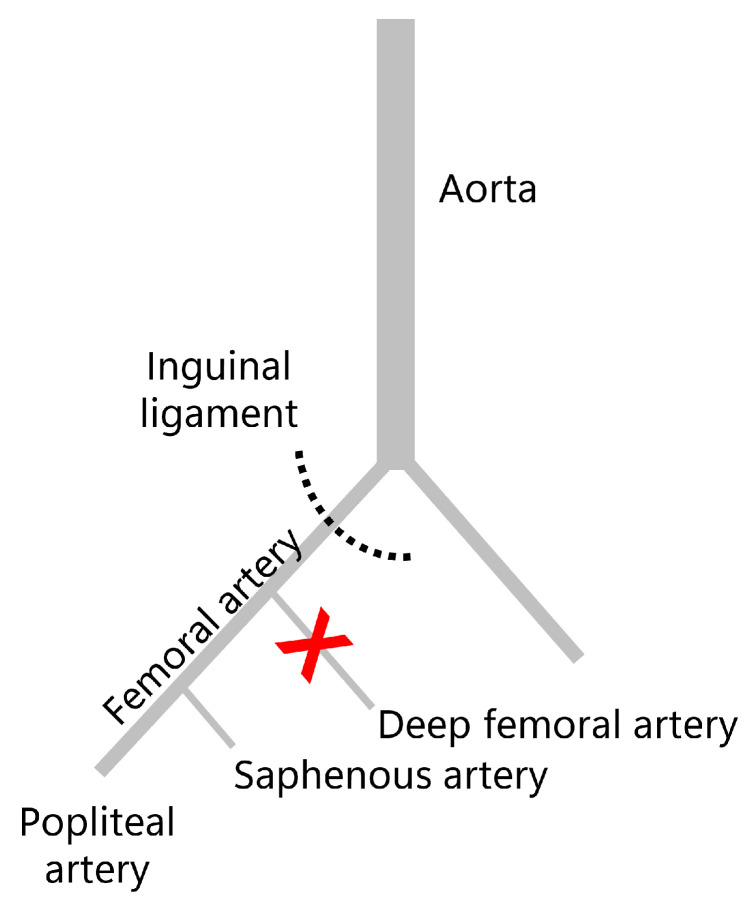
Murine ischemia model of fracture healing. Schematic drawing of the induced ischemia (X) at the deep femoral artery branching distally of the inguinal ligament along the femoral artery.

We used a well-established mild ischemia model, as previously described in detail [13]. To anesthesize the mice, intraperitoneal administration of 75 mg/kg body weight of ketamine (Pharmacia, Erlangen, Germany) and 15 mg/kg body weight of xylazine 2% (Bayer, Leverkusen, Germany) was used. The skin incision was made on the right hind limb medial to the patella in the direction of the femoral artery with a length of 6 mm parallel to the course of the vessel. In order to create ischemic conditions, the deep femoral artery was ligated twice with a non-absorbable 6-0 suture (Ethicon, Raritan, NJ, USA) (Figure 1). Firstly, the knee joint capsule was opened along the medial border of the patella in a longitudinal direction. This allowed the patella to be dislocated laterally, exposing the femoral condyles. Secondly, after drilling a 0.5 mm-diameter-hole into the intercondylar notch, a 0.4 mm-diameter injection needle was inserted into the intramedullary canal. Thereafter, a tungsten guidewire (0.2 mm in diameter) was inserted via the needle into the intramedullary canal. Once the needle has been removed, the femur was then fractured using a blunt guillotine at a defined weight and height. The fracture was stabilized by implanting an intramedullary titanium screw (MouseScrew™, RISystem, Davos, Switzerland) over the guidewire [14]. Radiography was then used to confirm the fracture and implant position.

Animals of the clopidogrel group (n = 24) received 3 mg/kg body weight of clopidogrel (Aliud Pharma GmbH, Laichingen, Germany) daily per os from the day of surgery as this dose has been shown to inhibit platelet aggregation in humans to a similar extent as 75 mg [15]. Animals of the control group (n = 22) received the equivalent volume of the vehicle (0.9% NaCl; Braun, Melsungen, Germany) by daily oral administration. The animals were sacrificed either 2 weeks (n = 15 in the clopidogrel group and n = 14 in the control group) or 5 weeks (n = 9 in the clopidogrel group and n = 8 in the control group) postoperatively by cervical dislocation. X-rays of each operated femur were taken immediately pre-sacrifice to exclude secondary dislocation of the implants. Subsequently, femurs were harvested and used for further analyses.

### 2.3. Biomechanical Analysis

For biomechanical analysis, the right and left femurs were resected at 2 weeks (n = 7 in the clopidogrel group and n = 8 in the control group) and 5 weeks (n = 9 in the clopidogrel group and n = 8 in the control group) and released from soft tissue. Callus stiffness was assessed [N/mm] using a three-point bending device after the implants were removed (Mini-Zwick Z 2.5; Zwick, Ulm, Germany) [16,17]. To obtain the relative bending stiffness [%], the results of the injured (right) side were compared with the bending stiffness of the healthy contralateral (left) femur (n = 9 in the clopidogrel group and n = 8 in the control group). Furthermore, this non-destructive approach for biomechanical analyses allows for the femurs to be used for subsequent micro-computed tomography (µCT) and histological investigations. Therefore, in line with the 3R principle (replacement, reduction, refinement), the number of animals required could be considerably reduced.

### 2.4. Radiological Analysis

X-rays (MX-20 Faxitron; X-ray Corporation, Wheeling, IL, USA) of the fractured femurs were performed at 2 weeks (n = 9 in the clopidogrel group and n = 8 in the control group) and 5 weeks (n = 9 in the clopidogrel group and n = 8 in the control group) after surgery to assess fracture healing by using the Goldberg score [18]. Fractured femurs were also analyzed by µCT (Skyscan 1176; Bruker, Billerica, MA, USA). For this purpose, the femurs were scanned at a spatial resolution of 9 μm with a standardized setup, as described previously [16,17]. Firstly, calcium hydroxyapatite (CaHA) phantom rods with known BMD (bone mineral density) values were used for calibration to express gray values as mineral content. Secondly, the region of interest (ROI) was contoured manually on each transversal slide. Here, exclusively novel bone was defined while excluding original cortical bone. The ROI was processed using a threshold procedure (CTAnalyzer, Bruker), which enabled differentiation between bone and soft tissue. The thresholds used to differentiate between bone and soft tissue were determined through visual inspection of the images, qualitative comparison with histological sections and analysis of previous µCT studies investigating bone repair and callus tissue [16,17]. Total mineralized bone was defined as a BMD of more than 0.410 g/cm^3^, equating to grey values of 68–255. For each specimen, the following µCT parameters were calculated from the callus ROI for each specimen: ratio of bone volume (BV) to total volume (TV) of the callus (BV/TV; [%]), trabecular number (TbN; [1/mm]), trabecular separation (TbSp; [mm]) and trabecular thickness (TbTh; [mm]).

### 2.5. Histomorphometric Analysis

Following the biomechanical and radiological analysis, the bones were fixed in formalin solution (4%, Carl Roth GmbH & Co. KG, Karlsruhe, Germany) for 24 h and then decalcified using a 10% ethylenediaminetetraacetate (EDTA) solution for 2 weeks. Longitudinal sections with a thickness of 5 µm were stained with Safranin-O (bone: turquoise, cartilage: red, cell nuclei: black, connective tissue: pale/pink) after embedding decalcified bones in paraffin (clopidogrel group: n = 8 at 2 weeks and n = 9 at 5 weeks; control group: n = 8 at each time point). As a basis for quantitative analysis, the histological specimens were digitized at 1.25× magnification (Keyence BioZero BZ8100 fluorescence microscope; Keyence Deutschland, Neu-Isenburg, Germany). The following structural indices were calculated in accordance with the recommendations of Gerstenfeld et al. [19]: (i) total periosteal callus area (CAr) [mm^2^], (ii) area of total osseous tissues (TOTAr)/CAr [%], (iii) area of cartilage (CgAr)/CAr [%], and (iv) area of fibrous tissue (FTAr)/CAr [%]. The total periosteal callus area was defined as the entire area of osseous, cartilaginous and fibrous callus tissue located outside of the cortices. The ImageJ Analysis System (1.54f, NIH, Bethesda, MD, USA) was used to mark and calculate each area. Furthermore, a system of scoring was employed to assess the quality of fracture bridging, as previously outlined [17].

### 2.6. Western Blot Analysis

The expression of proteins within the callus tissue was examined using Western blot analyses. This included the expression of bone morphogenetic protein (BMP)-2, BMP-4, CD31, cystein-rich angiogenic inducer (Cyr) 61, runt related transcription factor 2 (RUNX2) and proliferating cell nuclear antigen (PCNA). Two weeks after surgery, the callus was harvested (n = 6 in each group) and immediately stored in liquid nitrogen at −80 °C. Following storage of the total protein fraction, the proteins were separated and transferred to membranes. They were then probed with anti-BMP-2 and anti-BMP-4 (both R&D Systems, Minneapolis, MN, USA), anti-CD31 (Cell Signaling Technology Europe B.V., Frankfurt am Main, Germany), anti-Cyr61 (R&D Systems), anti-RUNX2 (Abcam, Cambridge, UK) and anti-PCNA (Proteintech, Planegg-Martinsried, Germany) antibodies. All antibodies were first incubated overnight at 4 °C, and then for a further 4 h at room temperature. The appropriate peroxidase-conjugated anti-IgG antibodies were employed as secondary antibodies (Dako Agilent Technologies, Carpinteria, CA, USA and R&D Systems). Protein expression was visualized via luminol-enhanced chemiluminescence after the membrane was exposed to the Intas ECL Chemocam Imager (Intas Science Imaging Instrument GmbH, Göttingen, Germany). To correct for unequal loading, signals were normalized to β-actin signals (Santa Cruz Biotechnology, Heidelberg, Germany).

### 2.7. Statistical Analysis

All statistical analyses were carried out using the SigmaPlot 13.0 software (Systat Software, Incorporated, San José, CA, USA). All data are given as means ± standard error of the mean (SEM) (95%-confidence interval and effect size in Supplementary Table S1). Data were first tested for normal distribution (Shapiro–Wilk test) and equal variances (F-test). An unpaired Student’s *t*-test was used to compare two experimental groups in case of parametric data. A Mann–Whitney U-test was performed in case of non-parametric distribution. A *p*-value < 0.05 was considered to indicate significant differences.

## 3. Results

### 3.1. Biomechanical Analysis

**Figure 2:**
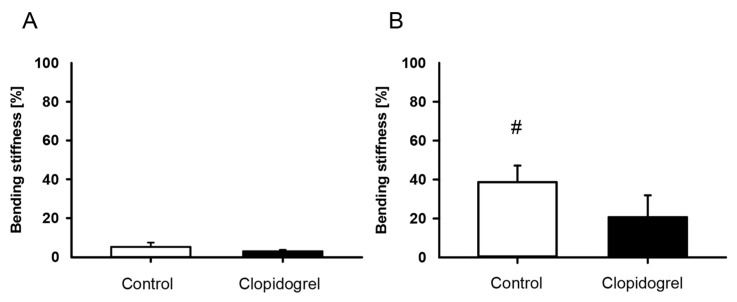
Biomechanical analysis of mouse femurs. (**A**,**B**): Ratio of bending stiffness of fractured to unfractured control (white bar; n = 8) and clopidogrel (black bar; n = 7/9 fractured/unfractured at 2 weeks, n = 9 at 5 weeks) femurs at 2 weeks (**A**) and 5 weeks **(B**) after surgery. Mean ± SEM. # *p* < 0.05 vs. control at 2 weeks.

The bending stiffness of fractured femurs of both groups was low at 2 weeks after surgery. Of interest, femurs of the clopidogrel group showed no significant increase in bending stiffness between 2 and 5 weeks after surgery in intragroup comparisons. In contrast, relative bending stiffness of femurs of the control group increased significantly over time (Figure 2). Intergroup comparisons between clopidogrel-treated and control animals did not show any significant differences in bending stiffness at 2 weeks and 5 weeks. Moreover, the healthy femurs of the contralateral side showed no difference between the clopidogrel and the control group after 2 weeks (clopidogrel: 121.4 ± 9.6 N/mm; control: 117.1 ± 13.2 N/mm) and 5 weeks (clopidogrel: 126.5 ± 16.4 N/mm; control: 117.0 ± 9.4 N/mm). Accordingly, relative bending stiffness did not show significant differences between both groups at 2 weeks (Figure 2A) and 5 weeks (Figure 2B), although a trend towards a lower relative bending stiffness could be observed in the clopidogrel group at 5 weeks after surgery.

### 3.2. Radiological Analysis

**Figure 3:**
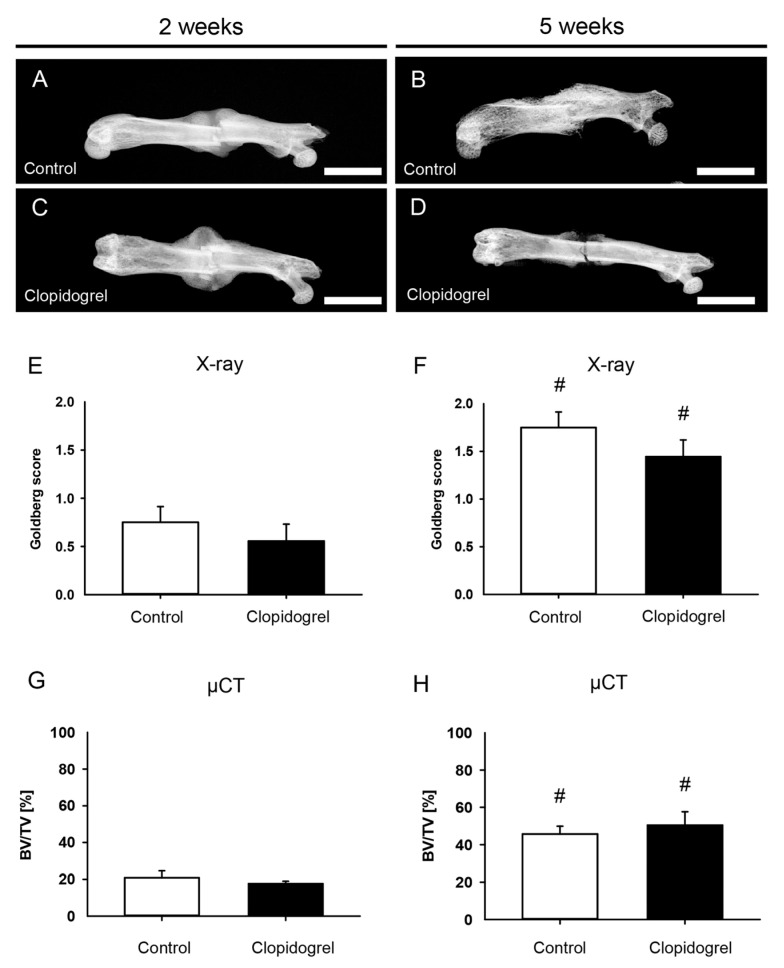
Radiological analysis (X-ray and µCT) of mouse femurs. (**A**–**D**): Representative X-ray images of femurs at 2 weeks (**A**,**C**) and 5 weeks (**B**,**D**) after surgery of control (**A**,**B**) and clopidogrel (**C**,**D**) animals. Scale bars: 2000 µm. (**E**,**F**): Goldberg score at 2 weeks (**E**) and 5 weeks (**F**) after surgery of control (white bar; n = 8) and clopidogrel (black bar; n = 9) animals. (**G**,**H**): Ratio of bone volume to tissue volume (BV/TV) at 2 weeks (**G**) and 5 weeks (**H**) after surgery within the callus of control (white bar; n = 8) and clopidogrel (black bar; n = 9) animals. Mean ± SEM. # *p* < 0.05 vs. clopidogrel/control at 2 weeks.

The X-rays of fractured femurs demonstrated a radiopaque callus formation at 2 weeks in both groups (Figure 3). The fracture gap was almost completely covered in the control group at 5 weeks after the operation, whereas the callus of clopidogrel animals at 5 weeks demonstrated no clear bridging (Figure 3A–D). The Goldberg score at 2 weeks postoperatively was low in both groups as a sign of incomplete osseous bridging and did not show significant differences between the groups (Figure 3E). The score increased significantly in both groups over time but did not show significant differences at 5 weeks after surgery (Figure 3F). In line with the X-ray analysis, µCT revealed no significant differences between femurs of the clopidogrel group compared to controls for BV/TV at 2 weeks and 5 weeks, while intragroup analyses showed a significant increase throughout the study period (Figure 3G,H). µCT analyses of the trabecular structures did not reveal significant differences between both study groups for TbN (2 weeks: control: 2.3 ± 0.4/mm, clopidogrel: 2.0 ± 0.1/mm; 5 weeks: control: 2.6 ± 0.3/mm, clopidogrel: 3.0 ± 0.3/mm), TbSp (2 weeks: control: 0.5 ± 0.1 mm, clopidogrel: 0.5 ± 0.0 mm; 5 weeks: control: 0.3 ± 0.0 mm, clopidogrel: 0.2 ± 0.0 mm) and TbTh (2 weeks: control: 0.1 ± 0.0 mm, clopidogrel: 0.1 ± 0.0 mm; 5 weeks: control: 0.2 ± 0.0 mm, clopidogrel: 0.2 ± 0.0 mm).

### 3.3. Histomorphometric Analysis

**Figure 4:**
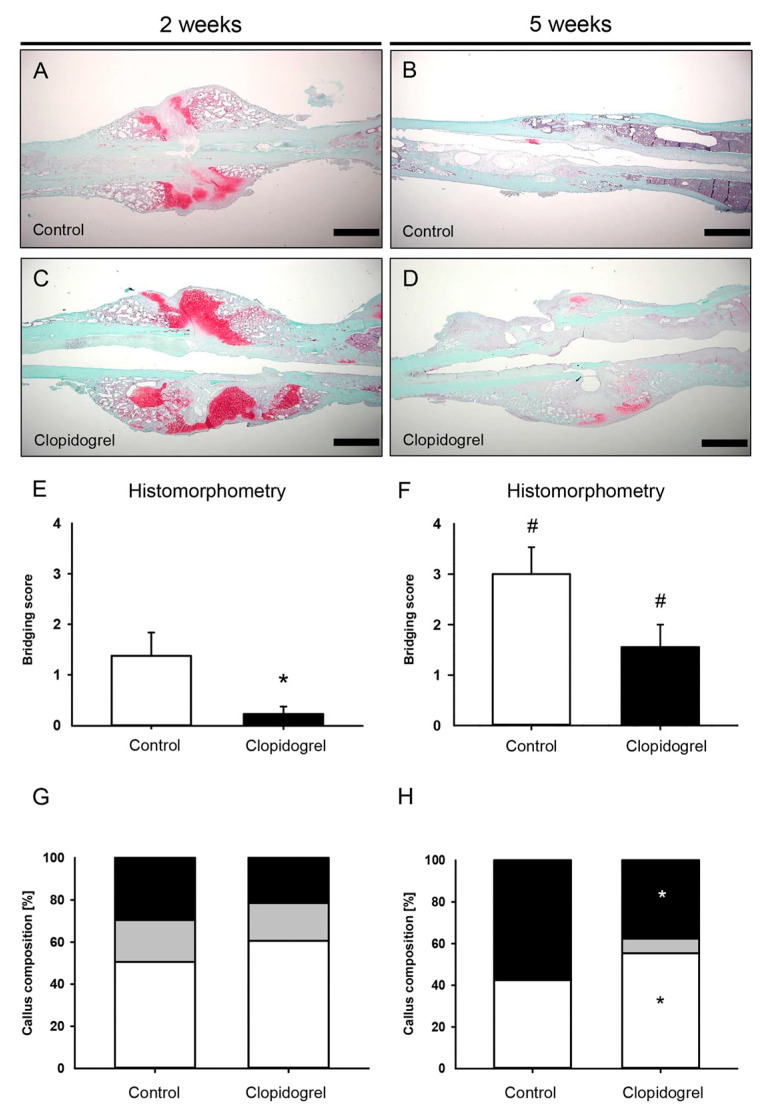
Histomorphometric analysis of mouse femurs. (**A**–**D**): Representative histological images of Safranin-O-stained femurs at 2 weeks (**A**,**C**) and 5 weeks (**B**,**D**) after surgery of control (**A**,**B**) and clopidogrel (**C**,**D**) animals. Scale bars: 500 µm. (**E**,**F**): Bridging score at 2 weeks (**E**) and 5 weeks (**F**) after surgery of control (white bar; n = 8) and clopidogrel (black bar; n = 8/9) animals. (**G**,**H**): Callus composition. Fraction of osseous callus (black), cartilaginous callus (gray) and connective tissue (white) of the total callus area of control (right bar; n = 8) and clopidogrel (left bar; n = 8/9) animals at 2 weeks (**G**) and 5 weeks (**H**) after surgery. Mean ± SEM; * *p* < 0.05 vs. control; # *p* < 0.05 vs. clopidogrel/control at 2 weeks.

This histomorphometric analysis of the fractured femurs at 2 weeks after surgery showed a callus formation at the site of injury that was lacking osseous bridging of the fracture in both groups at this early time point (Figure 4A,C). At 5 weeks after surgery, the callus appeared to be smaller in both groups as a typical sign of callus remodeling with signs of incomplete osseous bridging in animals of the clopidogrel group (Figure 4B,D). Accordingly, the CAr at 2 weeks was 10.3 ± 1.1 mm^2^ for control animals and 11.9 ± 1.0 mm^2^ for clopidogrel animals, whereas the CAr at 5 weeks was 5.0 ± 0.7 mm^2^ for controls and 5.5 ± 0.8 mm^2^ for clopidogrel animals (*p* > 0.05 in intergroup comparison). The bridging score was significantly reduced in clopidogrel animals at 2 weeks after surgery (Figure 4E). At 5 weeks after surgery, the bridging score did not show a significant difference between clopidogrel animals and controls (*p* = 0.054; Figure 4F). Analysis of tissue composition of the callus in clopidogrel animals at 5 weeks after surgery revealed a reduced TOTAr/CAr (control: 56.8 ± 4.9%, clopidogrel: 37.7 ± 4.6%) and, vice versa, an increased FTAr/CAr (control: 42.5 ± 5.0%, clopidogrel: 55.4 ± 4.1%) compared to controls, while no differences were found at 2 weeks after surgery (Figure 4G,H).

### 3.4. Western Blot Analysis

**Figure 5:**
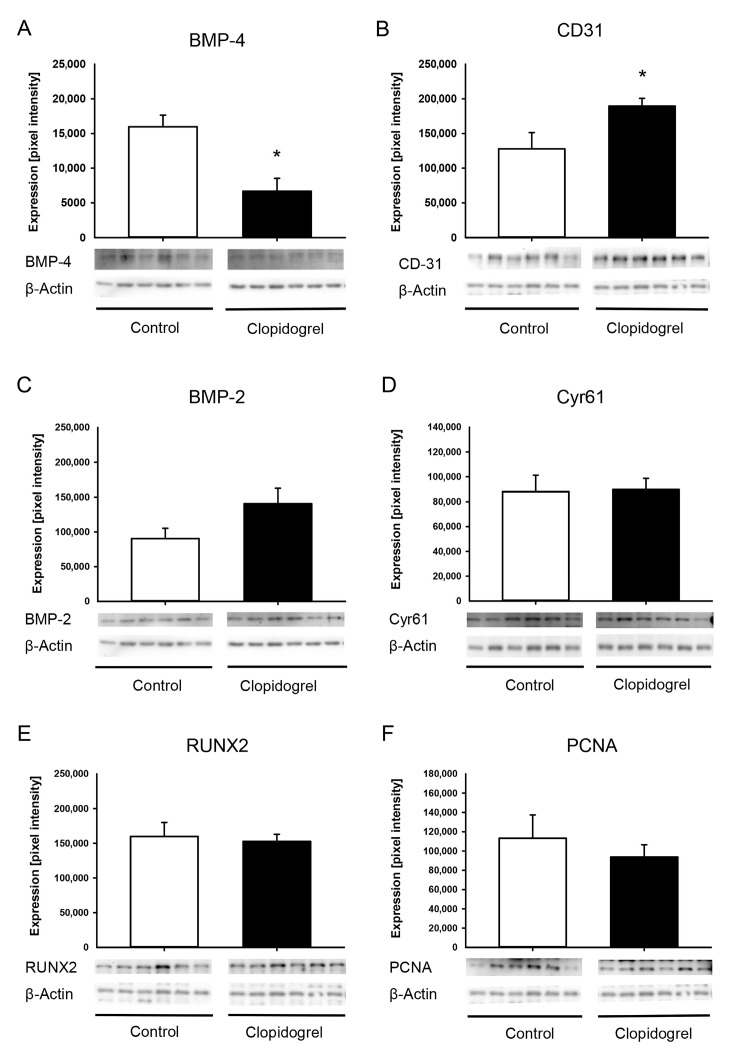
Western blot analysis of callus tissue. (**A**–**F**): Western blots and expression of BMP-4 (**A**), CD31 (**B**), BMP-2 (**C**), Cyr61 (**D**), RUNX2 (**E**), PCNA (**F**) and β-actin within the callus tissue of control (white bar; n = 6) and clopidogrel (black bar; n = 6) femurs at 2 weeks after surgery. Mean ± SEM; * *p* < 0.05 vs. control.

The Western blot analysis at 2 weeks after surgery revealed a significantly reduced expression of the osteogenic marker BMP-4 in the clopidogrel group compared to controls (Figure 5A). The blood vessel marker CD31 showed a significantly increased expression in clopidogrel animals compared to control animals (Figure 5B). The osteogenic markers BMP-2 and RUNX2 as well as the angiogenic marker Cyr61 and the proliferation marker PCNA did not differ between clopidogrel and control animals (Figure 5C–F).

A detailed statistical overview of all the collected measured values, including effect sizes and confidence intervals, can be found in Supplementary Table S1.

## 4. Discussion

The present study demonstrates for the first time that clopidogrel may impair fracture healing under ischemic conditions in a well-established murine model of delayed fracture healing.

Clopidogrel is a widely used drug in clinical practice and is often prescribed as a permanent medication by inhibiting the P_2_Y_12_ receptor [20,21]. This receptor is not only found on platelets, but also on osteoblasts and osteoclasts [6,22,23]. Previous studies have assessed the impact of clopidogrel on bone healing, yielding conflicting results. Syberg et al. [8] could show that clopidogrel has a negative effect on bone density and trabecular properties in vivo of female ovariectomized mice and reported for the first time the inhibition of osteoblast and osteoclast function by clopidogrel in vitro. Additionally, they demonstrated that clopidogrel causes an increased differentiation of mesenchymal precursor cells from osteoblasts to adipocytes in cell cultures. In line with Syberg et al. [8], histomorphometric analyses of the present study demonstrated a significantly lower proportion of bone tissue and an increased proportion of connective tissue in the total callus area at 5 weeks after surgery, indicative of a delayed remodeling process under the influence of clopidogrel at this stage of the healing time point. Previous studies have shown that reliable femoral fracture consolidation occurs five weeks after surgery [24,25]. However, in this well-established ischemia model, ischemia delays the process of bone regeneration [13]. Therefore, the assessment of clopidogrel under ischemic conditions over a longer period of time could be of interest. The reduced osseous bridging score as well as the biomechanical results throughout the observation period are additional indicators for a delayed bone healing process in animals treated with clopidogrel. The different results of the radiological analysis in the present study compared to the results of Syberg et al. [8] may be due to a different dosage of clopidogrel applied to the animals (3 mg/kg vs. 1 mg/kg body weight). Although the radiological and biomechanical intergroup analyses revealed no significant differences, the histomorphometric and Western blot analyses showed statistically significant impairments in the clopidogrel group. These discrepancies could be due to resolution limits, sensitivity to subtle changes or differential remodelling dynamics. Furthermore, despite the lack of statistical significance, clear differences were observed in the intergroup analysis five weeks after surgery (e.g., Figure 2B and Figure 3F).

In contrast, Lillis et al. [6] demonstrated that clopidogrel has a positive influence on the regeneration of bone defects in rabbit skulls. These controversial results compared to the present study may be attributed to a variety of factors, such as the treatment regime of clopidogrel, the induced ischemia and the conditions at the time of injury to the bone. While Lillis et al. [6] began clopidogrel treatment 1 week prior to surgery and induced a bone defect under physiological healing conditions, fractures in the present study were induced under ischemic conditions and clopidogrel administration commenced on the day of surgery. Moreover, ischemia was induced simultaneously to the fracture so that no formation of collateral circulation could take place that might have potentially attenuated ischemic conditions. In addition, the pharmacological effect of clopidogrel on cells with P_2_Y_12_ receptors set in after the injury during the healing process instead of preconditioning drug administration. These factors may have significantly influenced the effect of clopidogrel on fracture healing. Hence, follow-up studies with different application times and dose-dependent investigations of clopidogrel should investigate its effect on fracture healing under different conditions in more detail. Fracture healing is a well-orchestrated series of events in which angiogenesis and osteogenesis are crucially important and closely linked to each other [26,27,28]. For successful bone healing, osteogenesis follows angiogenesis throughout the healing process [26].

Angiogenesis is known to be influenced by several factors such as e.g., CD31 and hypoxia [5,29]. CD31 is known to have both a stabilizing effect on existing vessels by mediating endovascular cell–cell contacts and an angiogenic effect, especially in ischemic tissue [5,30]. Under physiological conditions, CD31 has the potential to accelerate bone formation by increasing the formation of vessels in the zone of bone regeneration [31]. Moreover, hypoxia is known to be a critical driving force for angiogenesis triggering the osteogenic-angiogenic coupling [30]. The increased expression of CD31 and ischemia-induced hypoxia in animals after treatment with clopidogrel in the present study may have, therefore, improved angiogenesis at the fracture site. However, this may not necessarily lead to improved osteogenesis.

Among others, osteogenesis is regulated by BMPs, which are known to have an osteogenic effect [32,33,34]. BMP-4 signaling plays a pivotal role in regulating the functions of osteoblasts, such as their differentiation and their production of extracellular matrix proteins (e.g., collagen and osteocalcin) and, thereby, improves the survival of osteoblasts [32,34,35,36,37]. These cells, in turn, are crucial for the expression of BMP-4 in bone [8]. In line with these findings, the reduced expression of BMP-4 in the callus of clopidogrel-treated animals may have contributed to the reduced fraction of bone in the callus composition. It may be speculated that interrupting an osteoblast-mediated positive feedback contributed to the detrimental effect of clopidogrel on the process of fracture healing at five weeks. Further studies are needed to investigate the precise impact of clopidogrel on the BMP-4 signaling pathway during bone healing.

The increased expression of CD31 and the decreased expression of osteogenic BMP-4 may lead to an altered ratio of angiogenesis to osteogenesis in the callus tissue of clopidogrel-treated animals. Of interest, previous studies have demonstrated that a pro-angiogenic shift in this ratio has a negative effect on fracture healing [38,39,40]. In fact, Orth et al. [38] could demonstrate that a strong angiogenic stimulus by local application of microvascular fragments can even impair fracture healing under physiological conditions. In turn, the spatiotemporally controlled administration of growth factors (VEGF:BMP-2 (1:2); pro-osteogenic ratio of angiogenesis/osteogenesis) has been shown to improve bone healing in a challenging murine non-union model [39]. In line with these observations, the impaired bone healing after treatment with clopidogrel may have been a result of a pro-angiogenic shift in angiogenic and osteogenic expression markers within the callus.

## 5. Conclusions

The present study demonstrates that clopidogrel may impair fracture healing under challenging ischemic conditions in a well-established murine fracture model. As impaired healing may lead to severe cases of non-union, further studies should investigate whether clopidogrel influences the process of bone healing also under non-ischemic conditions. Therefore, future translational studies are necessary to assess whether these findings may help in clinical treatment.

## Data Availability

The original contributions presented in the study are included in the article/Supplementary Materials, further inquiries can be directed to the corresponding author.

## Supplementary Materials

The following supporting information can be downloaded at: https://www.mdpi.com/article/10.3390/biomedicines13092286/s1, Table S1: Overview of all the collected measured values including means, standard error of the mean, 95%-confidence intervals for mean, *p*-values (intergroup comparison) and Cohen’s d. Bold type, if *p* < 0.05.

## Abbreviations

The following abbreviations are used in this manuscript:

BMP	Bone Morphogenetic Protein
PAD	Peripheral Arterial Occlusive Disease
CLI	Critical Limb Ischemia
NIH	National Institutes of Health
µCT	Micro-Computed Tomography
BMD	Bone Mineral Density
CaHA	Calcium Hydroxyapatit
ROI	Region Of Interest
BV	Bone Volume
TV	Total Volume
TbN	Trabecular Number
TbSp	Trabecular Separation
TbTh	Trabecular Thickness
EDTA	Ethylenediaminetetraacetate
Cyr	Cystein-rich angiogenic inducer
RUNX	Runt related transcription factor
PCNA	Proliferating Cell Nuclear Antigen
SEM	Standard Error of the Mean
