# Supplementary material for: Clopidogrel Influences Fracture Healing Under Ischemic Conditions
Source: Biomedicines. 2025 Sep 17;13(9):2286. doi: 10.3390/biomedicines13092286 (PMC12467816; doi:10.3390/biomedicines13092286)
Supplement: Supplementary file 1 [file biomedicines-13-02286-s001.zip › biomedicines-3795812-supplementary.pdf]

	means ± standard error of the mean	95%-confidence intervals for mean	p-value (intergroup comparison)	Cohen's d
**Biomechanical analysis**				
bending stiffness (control/2 weeks)	4.7 ± 1.6 N/mm	[0.8-8.6]	0.522	0.32
bending stiffness (clopidogrel/2weeks)	3.5 ± 0.9 N/mm	[1.3-5.7]		
bending stiffness (control/5 weeks)	43.0 ± 9.0 N/mm	[21.7-64.3]	0.152	0.60
bending stiffness (clopidogrel/5weeks)	24.7 ± 12.9 N/mm	[-6.7-56.1]		
relative bending stiffness (control/2 weeks)	5.2 ± 2.2%	[0.0-10.4]	0.867	0.49
relative bending stiffness (clopidogrel/2weeks)	2.9 ± 0.7%	[1.1-4.7]		
relative bending stiffness (control/5 weeks)	38.7 ± 8.5%	[18.7-58.7]	0.152	-0.016
relative bending stiffness (clopidogrel/5weeks)	39.3 ± 15.1%	[4.6-74.0]		
bending stiffness contralateral (control/2weeks)	117.1 ± 13.2 N/mm	[85.9-148.3]	0.795	-0.13
bending stiffness contralateral (clopidogrel/2weeks)	121.4 ± 9.6 N/mm	[99.3-143.5]		
bending stiffness contralateral (control/5weeks)	117.0 ± 9.4 N/mm	[94.8-139.2]	0.451	-0.26
bending stiffness contralateral (clopidogrel/5 weeks)	126.5 ± 16.4 N/mm	[86.4-166.6]		
**Radiological analysis**				
Goldberg score (control/2 weeks)	0.8 ± 0.2	[0.4-1.2]	0.451	1.11
Goldberg score (clopidogrel/2 weeks)	0.6 ± 0.2	[0.2-1.0]		
Goldberg score (control/5 weeks)	1.8 ± 0.2	[1.4-2.2]	0.237	1.81
Goldberg score (clopidogrel/5 weeks)	1.4 ± 0.2	[1.0-1.8]		
BV/TV (control/2 weeks)	21.0 ± 3.7%	[12.3-29.7]	0.400	0.433
BV/TV (clopidogrel/2 weeks)	17.7 ± 1.3%	[14.8-20.6]		
BV/TV (control/5 weeks)	45.7 ± 4.2%	[35.8-55.6]	0.590	-0.265
BV/TV (clopidogrel/5 weeks)	50.4 ± 7.2%	[33.8-67.0]		

TbN (control/2 weeks)	2.3 ± 0.4/mm	[1.3-3.3]	0.810	0.361
TbN (clopidogrel/2 weeks)	2.0 ± 0.1/mm	[1.8-2.2]		
TbN (control/5 weeks)	2.6 ± 0.3/mm	[1.9-3.3]	0.434	-0.443
TbN (clopidogrel/5 weeks)	3.0 ± 0.3/mm	[2.3-3.7]		
TbSp (control/2 weeks)	0.5 ± 0.1mm	[0.3-0.7]	1.000	0.000
TbSp (clopidogrel/2 weeks)	0.5 ± 0.0mm	[0.46-0.54]		
TbSp (control/5 weeks)	0.3 ± 0.0mm	[0.2-0.4]	0.590	0.866
TbSp (clopidogrel/5 weeks)	0.2 ± 0.0mm	[0.1-0.3]		
TbTh (control/2 weeks)	0.1 ± 0.0mm	[0.08-0.12]	0.911	0.000
TbTh (clopidogrel/2 weeks)	0.1 ± 0.0mm	[0.08-0.12]		
TbTh (control/5 weeks)	0.2 ± 0.0mm	[0.17-0.23]	0.618	0.000
TbTh (clopidogrel/ 5 weeks)	0.2 ± 0.0mm	[0.15-0.25]		
**Histomorphometric analysis**				
Bridging score (control/2 weeks)	1.4 ± 0.5	[0.3-2.5]	**0.024**	1.248
Bridging score (clopidogrel/2 weeks)	0.2 ± 0.2	[-0.1-0.5]		
Bridging score (control/5 weeks)	3.0 ± 0.5	[1.7-4.3]	0.053	1.002
Bridging score (clopidogrel/5 weeks)	1.6 ± 0.4	[0.6-2.6]		
CAr (control/2 weeks)	10.3 ± 1.1 mm^2	[7.7-12.9]	0.317	-0.525
CAr (clopidogrel/2 weeks)	11.9 ± 1.0 mm^2	[9.4-14.4]		
CAr (control/5 weeks)	5.0 ± 0.7 mm^2	[3.3-6.7]	0.670	-0.215
CAr (clopidogrel/5 weeks)	5.5 ± 0.8 mm^2	[3.5-7.5]		
TOTAr/CAr (control/2 weeks)	29.6 ± 4.7%	[18.4-40.8]	0.183	0.695
TOTAr/CAr (clopidogrel/2 weeks)	21.5 ± 3.4%	[13.5-29.5]		
TOTAr/CAr (control/5 weeks)	56.8 ± 4.9%	[45.2-68.4]	**0.012**	1.389
TOTAr/CAr (clopidogrel/5 weeks)	37.7 ± 4.6%	[27.2-48.2]		

CgAr/CAr (control/2 weeks)	19.8 ± 6.7%	[3.9-35.7]	0.832	0.111
CgAr/CAr (clopidogrel/2 weeks)	17.9 ± 5.3%	[5.3-30.5]		
CgAr/CAr (control/5 weeks)	0.7 ± 0.4%	[-0.7-1.7]	0.389	-0.979
CgAr/CAr (clopidogrel/5 weeks)	6.9 ± 2.9%	[0.3-13.5]		
FTAr/CAr (control/2 weeks)	50.6 ± 6.5%	[45.2-66.0]	0.318	-0.518
FTAr/CAr (clopidogrel/2 weeks)	60.6 ± 7.1%	[43.8-77.4]		
FTAr/CAr (control/5 weeks)	42.5 ± 5.0%	[30.7-54.3]	**0.018**	-0.979
FTAr/CAr (clopidogrel/5 weeks)	55.4 ± 4.1%	[46.0-64.8]		
**Western blot analysis**				
BMP-4 (control/2weeks)	15962.8 ± 1688.1	[11623.4-20302.2]	**0.004**	2.138
BMP-4 (clopidogrel/2weeks)	6685.7 ± 1851.2	[4834.6-8536.9]		
CD31 (control/2 weeks)	127852.0 ± 23561.2	[67285.9-188418.1]	**0.039**	-3.144
CD31 (clopidogrel/2 weeks)	189576.4 ± 11086.5	[161077.7-218075.1]		
BMP-2 (control/2 weeks)	90469.1 ± 14849.5	[52297.3-128640.9]	0.087	-1.093
BMP-2 (clopidogrel/2 weeks)	140650.6 ± 21964.3	[84189.6-197111.6]		
Cyr61 (control/2 weeks)	88073.2 ± 13146.1	[54280.2-121866.2]	0.917	-0.061
Cyr61 (clopidogrel/2 weeks)	89763.5 ± 8919.9	[66834.1-112692.9]		
RUNX2 (control/2 weeks)	159718.4 ± 20249.4	[107665.7-211771.1]	0.382	0.178
RUNX2 (clopidogrel/5 weeks)	152726.6 ± 10267.3	[126333.7-179119.5]		
PCNA (control/2 weeks)	113199.5 ± 24231.0	[50911.6-175487.4]	0.493	0.411
PCNA (clopidogrel/5 weeks)	93744.8 ± 12691.1	[61121.3-126.368.3]		

Supplementary Table S1

Overview of all the collected measured values including means standard error of the mean, 95%-confidence intervals for mean, p-values (intergroup comparison) and Cohen's d. Bold type, if $p < 0.05$.
